# Developmental changes in electrophysiological properties of auditory cortical neurons in the *Cntnap2* knockout rat

**DOI:** 10.1152/jn.00029.2022

**Published:** 2023-03-22

**Authors:** Rajkamalpreet S. Mann, Brian L. Allman, Susanne Schmid

**Affiliations:** Department of Anatomy and Cell Biology, Schulich School of Medicine & Dentistry, University of Western Ontario, London, Ontario, Canada

**Keywords:** auditory cortex, auditory processing, autism, patch-clamp, development

## Abstract

Disruptions in the *CNTNAP2* gene are known to cause language impairments and symptoms associated with autism spectrum disorder (ASD). Importantly, knocking out this gene in rodents results in ASD-like symptoms that include auditory processing deficits. This study used in vitro patch-clamp electrophysiology to examine developmental alterations in auditory cortex pyramidal neurons of *Cntnap2^−/−^* rats, hypothesizing that *CNTNAP2* is essential for maintaining intrinsic neuronal properties and synaptic wiring in the developing auditory cortex. Whole cell patch-clamp recordings were conducted in wildtype and *Cntnap2^−/−^* littermates at three postnatal age ranges (*P8*–*12*, *P18*–*21*, and *P70*–*90*). Consistent changes across age were seen in all measures of intrinsic membrane properties and spontaneous synaptic input. Intrinsic cell properties such as action potential half-widths, rheobase, and action-potential firing frequencies were different between wildtype and *Cntnap2^−/−^* rats predominantly during the juvenile stage (*P18*–*21*), whereas adult *Cntnap2^−/−^* rats showed higher frequencies of spontaneous and mini postsynaptic currents (sPSCs; mPSCs), with lower sPSC amplitudes. These results indicate that intrinsic cell properties are altered in *Cntnap2^−/−^* rats during the juvenile age, leading to a hyperexcitable phenotype during this stage of synaptic remodeling and refinement. Although intrinsic properties eventually normalize by reaching adulthood, changes in synaptic input, potentially caused by the differences in intrinsic membrane properties, seem to manifest in the adult age and are presumably responsible for the hyperreactive behavioral phenotype. In conjunction with a previous study, the present results also indicate a large influence of breeding scheme, i.e., pre- or postnatal environment, on the impact of *Cntnap2* on cellular physiology.

**NEW & NOTEWORTHY** This study shows that neurons in the auditory cortex of *Cntnap2* knockout rats are hyperexcitable only during the juvenile age, whereas resulting changes in synaptic input persist in the adult. In conjunction with a previous study, the present results indicate that it is not the genes alone, but also the influence of pre- and postnatal environment, that shape neuronal function, highlighting the importance of early intervention in neurodevelopmental disorders.

## Graphical abstract

**Figure:**
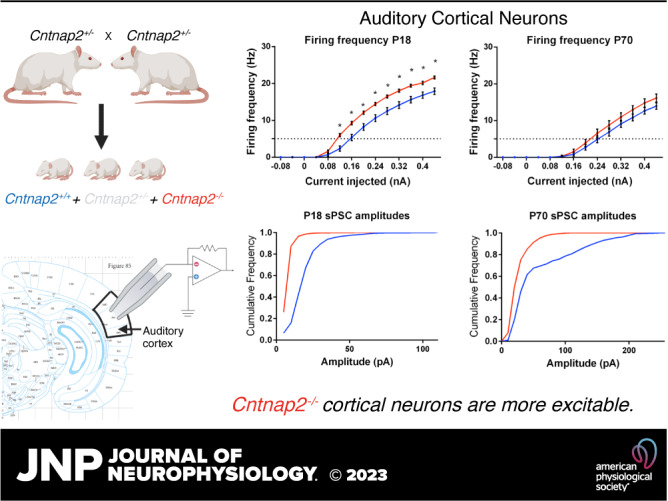

## INTRODUCTION

Disruptions during the critical periods of sensory system development can result in persistent alterations in behavior, often manifesting in developmental disorders. The contactin-associated protein-like-2 (*CNTNAP2*) gene is an important gene expressed predominantly during development and in sensory pathways (1, 2), and *CNTNAP2* mutations have been implicated in the onset of developmental disorders such as autism spectrum disorder (ASD) and developmental language disorder (1, 3–6). *CNTNAP2* encodes a transmembrane protein (CASPR2) that belongs to the neurexin family and is commonly known to localize and concentrate voltage-gated K+ channels at juxtaparanodes of myelinated axons, as well as to stabilize synapses (7, 8). Although recent studies on rodents have begun to characterize the cellular mechanisms associated with disruptions in *CNTNAP2* (9, 10), we still lack a complete understanding of how a functional loss of *CNTNAP2* alters cell properties and neuronal signaling during the development of sensory cortices.

Importantly, *CNTNAP2* is not exclusive to humans and is expressed throughout the cortico-striato-thalamic circuit in other animals, such as songbirds and rodents (11–13), suggesting the gene is highly conserved and is important for sensory processing at different brain levels. Indeed, a homozygous *Cntnap2* gene knockout in mice and rats is known to disrupt basic behavioral functions relying on auditory processing, such as the acoustic startle response and sensorimotor gating (13–16). These basic auditory processing functions involve established brainstem circuits as well as higher level cortical areas, but the neuronal changes in these circuits at the cellular level remain poorly understood, especially during the critical period of development of the auditory system. Thus, the objective of this study is to use a knockout rat model to investigate the impact of a loss of *Cntnap2* on the function of pyramidal neurons in the primary auditory cortex throughout development.

Recent studies have examined cortical pyramidal neurons from *Cntnap2* knockout mice in cell cultures and found that the functional and morphological properties of these neurons were altered. Specifically, the loss of *Cntnap2* resulted in decreased spine density, altered glutamatergic synapses, altered AMPAr/NMDAr mediated excitatory and inhibitory postsynaptic potentials (EPSCs and IPSCs) and decreased dendritic arborization (17, 18); cell properties that are important for the proper relaying of neural information. Synaptic differences have been reported in pyramidal neurons of visual cortices of *Cntnap2*^−/−^ mice at older but not younger ages, as well as in medial prefrontal cortex (mPFC) (19, 20). Differences in intrinsic properties of inhibitory interneurons in the somatosensory cortex and mPFC have also been reported in *Cntnap2*^−/−^ mice (21, 22). However, given that *Cntnap2* is extensively involved in auditory processing (9, 14–16) and expressed in the auditory system (2, 23), it would be important to study cortical areas involved in auditory information processing. In a recent study from our laboratory, we reported marked differences in the intrinsic excitability, but no synaptic differences, in the pyramidal neurons of auditory cortices of adult *Cntnap2^−^*^/−^ rats (9). Given that a past study in *Cntnap2^−^*^/−^ mice visual cortices found that electrophysiological differences were not consistent across age (19), it is important to assess the impact of *Cntnap2* deletion in the auditory cortex throughout development.

To address this, we investigated the effect of a functional loss of *Cntnap2* on the intrinsic cellular properties and synaptic input of pyramidal cells over development in the rat auditory cortex in acute brain slices. Overall, we hypothesize that *Cntnap2* knockout differentially affects the intrinsic properties and excitability of auditory cortical neurons in different age stages (e.g., juveniles vs. adults). To examine the intrinsic properties, we measured resting membrane potential, membrane resistance and capacitance, and intrinsic excitability as assessed by action potential properties and threshold. Neuronal excitability was further assessed by recording action potential firing frequencies in response to increasing current stimuli. Synaptic input was assessed by recording spontaneous, miniature, and evoked excitatory postsynaptic potentials from pyramidal neurons in layers 2/3 of the auditory cortex.

## METHODS

### Animals

Male and female Sprague-Dawley wildtype and homozygous knockout (*Cntnap2^−^*^/−^) brain slices were obtained from litters resulting from heterozygous (*Cntnap2^+/−^*) crossings for littermate controls. Animals were used at three age ranges of *postnatal days 8*–*12* (*P8*–*12*, infant; wildtype: *n* = 3 rats, 11–14 cells; *Cntnap2*^−/−^: *n* = 4 rats, 20–22 cells), *P18*–*21* (juvenile; wildtype: *n* = 7 rats, 22–40 cells; *Cntnap2*^−/−^: *n* = 9 rats, 38–47 cells), and *P70*–*90* (adult, wildtype: *n* = 5 rats, 15–17 cells; *Cntnap2^−/−^*: *n* = 6 rats, 22–24 cells). Animals were obtained and bred as described in a comprehensive behavioral study conducted by Scott et al. (14). Briefly, original heterozygous *Cntnap2*^+/−^ breeders were obtained from Horizon Discovery (Boyertown, PA; originally created at SAGE Laboratories, Inc. in conjunction with Autism Speaks; the line is now maintained by Envigo), and experimental animals were obtained through the breeding colony maintained in our laboratory. Date of birth was designated as *postnatal day zero* (*P0*). Rats were genotyped either by toe clipping around *P5*–*7* (for animals to be used in *P8*–*12* and *P18*–*21* experiments), or ear punches at *P21* (for *P70*–*90* experiments). Rats were weaned on *P21*, and sexes were separated by *P35*. Rats were housed in a temperature-controlled room on a 12-h light/dark cycle, with ad libitum food and water, and were never housed alone unless necessary. All procedures were approved by the University of Western Ontario Animal Care Committee and were in accordance with the guidelines established by the Canadian Council on Animal Care.

### In Vitro Electrophysiological Recordings

#### Slice preparation.

Sprague-Dawley wildtype (*n* = 16) and homozygous *Cntnap2*^−/−^ (*n* = 19) rats were anesthetized with isofluorane and their brains quickly removed and transferred into ice-cold slicing solution containing (in mM): 2.5 KCl, 1.25 NaH_2_PO_4_-H_2_O, 24 NaHCO_3_, 10 MgSO_4_, 11 glucose, 234 sucrose, 2 CaCl_2_, 3 Myoinositol, 2 Na-Pyruvate, and 0.4 ascorbate; equilibrated with 95% O_2_/5% CO_2_. Coronal slices (3.7 and 4.5 mm caudal to bregma) of 300 µm thickness were cut with a vibrating microtome (Compresstome VF-200, Precisionary) in a chamber filled with ice-cold preparation solution, and subsequently transferred into a holding chamber filled with artificial cerebrospinal fluid (ACSF) containing (in mM): 3 KCl, 1.25 NaH_2_PO_4_-H_2_O, 3 MgSO_4_, 26 NaHCO_3_, 124 NaCl, and 10 glucose; equilibrated with 95% O_2_/5% CO_2_. CaCl_2_ (2 mM) was added to the ACSF a few minutes before slices were transferred. The ACSF containing the slices was heated to ∼35°C for 30 min, and the slices were left to rest for an additional 1 h at room temperature to recover. Slices were kept at room temperature during the experiment.

#### Whole cell recordings.

Electrophysiological experiments were performed as reported previously (24–26). In brief, whole cell patch-clamp electrophysiology of visually identified pyramidal neurons in layers 2/3 of the general auditory cortex (in variable locations of auditory cortex subfields) was conducted using an upright microscope (Zeiss Axioskop, Germany), equipped with an EMCCD camera (Evolve 512, Photometric, Tuscon, AZ). Recording electrodes were pulled on a P-97 Puller (Sutter Instrument, Novato, CA) from fabricated borosilicate glass capillaries (1B150F-4, OD: 1.50 mm, ID: 0.84 mm, World Precision Instruments, Sarasota, FL) and had 3–7 MΩ resistance when filled with an intracellular solution containing the following (in mM): 140 K-gluconate, 10 KCl, 1 MgCl_2_, 10 HEPES, 0.02 EGTA, 3 Mg-ATP, and 0.5 Na-GTP, pH adjusted to 7.3, 290–300 mosmol/kgH_2_O/L). Signals were sampled at 5 kHz, amplified with Axopatch 200B, digitized with Digidata1550, and analyzed using pClamp10.4 (all Axon Instruments, Molecular Devices, Sunnydale, CA). Only pyramidal cells with access resistance < 25 MΩ were included in analyses and parameters were monitored throughout recordings. The pipette capacitance was compensated, but the access resistance and cell capacitance were not compensated during recordings. Junction potential was not compensated but was calculated to be +15 mV using the Nernst–Planck equation using LJPcalc software (https://swharden.com/LJPcalc; 27).

**Figure 1.**
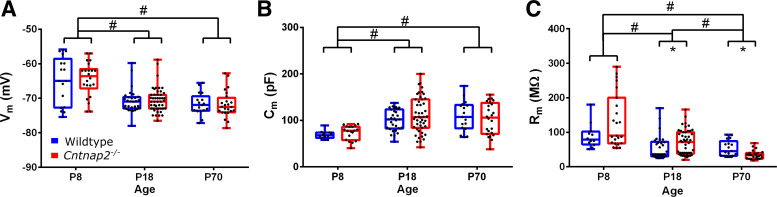
Passive membrane properties. Resting membrane potential (*A*), cell capacitance (*B*), and membrane resistance of auditory cortical neurons in wildtype (blue) and *Cntnap2^–/–^* rats (red) (*C*) at *postnatal age* (*P*)*8*–*12*, *P18*–*21*, and *P70*–*90*. Graphs show means ± SE. *Significant differences between genotypes *P* < 0.05. #Significant differences between age groups *P* < 0.05.

**Figure 2.**
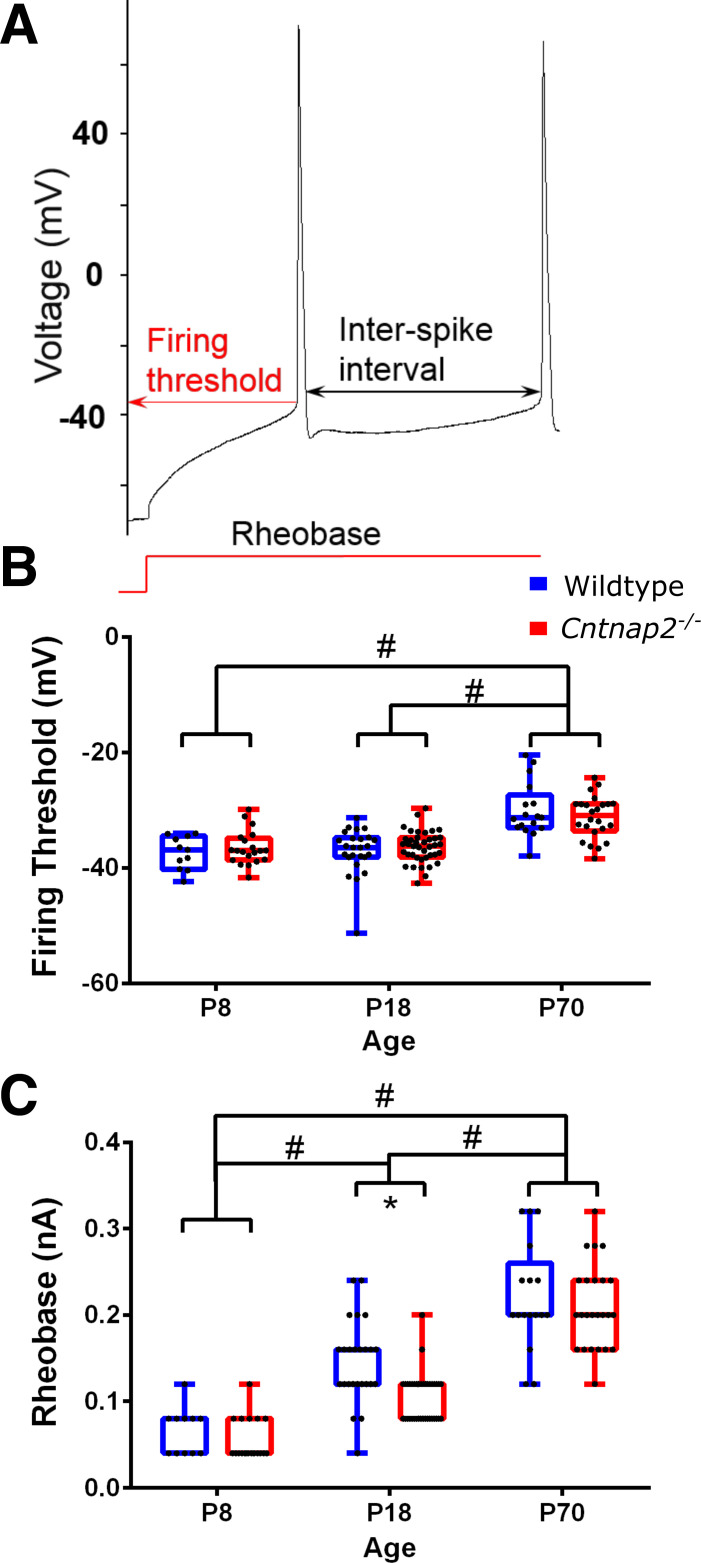
Action potential features. *A*: sample current clamp recording indicating the action potential features assessed. *B*: firing thresholds of action potentials in wildtype (blue) and *Cntnap2*^−/−^ neurons (red) at ages *postnatal* (*P*)*8*–*12*, *P18*–*21*, and *P70*–*90*, recorded by step current stimulations. *C*: rheobase currents. Graphs show means ± SE. *Significant differences between genotypes *P* < 0.05. #Significant differences between age groups *P* < 0.05.

**Figure 3.**
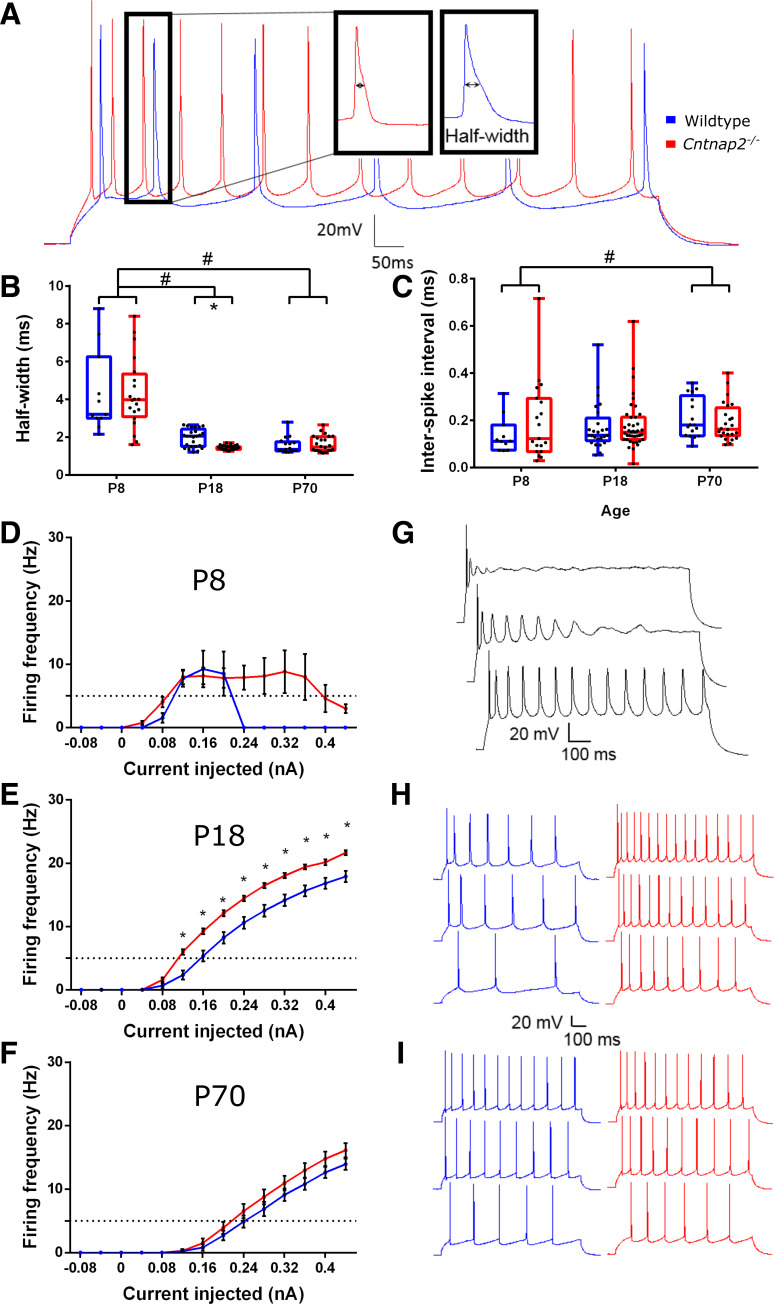
Action potential half-width and firing profiles. *A*: sample current clamp recording of action potentials from a *Cntnap2*^−/−^ and wildtype neuron in response to the same step current stimulation. *B*: action potential half-widths decrease during development, but *Cntnap2*^−/−^ (red) neurons exhibit smaller half-widths than wildtypes (blue) at *postnatal age* (*P*)*18*. *C*: interspike intervals (ISI) of action potentials show no significant changes during development or between genotypes. *D*–*I*: firing frequencies at different age stages as indicated and a sample firing recording in response to increasing current stimuli. Graphs show means ± SE. *Significant differences between genotypes, *P* < 0.05. #Significant differences between age groups, *P* < 0.05.

Voltage-clamp membrane test using a 10-mV step was used to assess cell capacitance and membrane resistance (Fig. 1), as well as other whole cell properties like access resistance. The membrane potential was held at −70 mV for all voltage-clamp recordings. Resting membrane potentials in wildtype and *Cntnap2^−^*^/−^ were measured in current-clamp while holding the current at *I* = 0. Current-clamp recordings to measure action-potentials were made while adjusting the current to keep the cells at −70 mV, which was close to their resting potentials, and involved 1-s long step current injections in 40 pA increments from −120 pA to +480 pA. These recordings were used to assess the firing threshold, rheobase, action potential half-width, interspike intervals, and firing rates (Figs. 2 and 3). Spontaneous postsynaptic currents (sPSCs) were assessed in voltage clamp by 5 min recordings of cell currents while holding the cells at −70 mV and mini post-synaptic currents (mPSCs) were similarly recorded but with the addition of 1 µM tetrodotoxin (TTX) in the bath. For the evoked PSCs, layers 5/6 of the auditory cortex were stimulated using a bipolar tungsten electrode (Science Products), and paired pulses were generated by a pulse generator (Master-8, AMPI, Israel) in wildtype and *Cntnap2^−^*^/−^ cells. This stimulation in *layers 5*/*6* likely activates a variety of synapses, including inputs from neurons in *layers 5* and *6*, axons from other areas, and antidromic stimulation of *layer 2*/*3* neurons. To determine paired-pulse ratios, interstimulus intervals of 20, 50, 100, 136, 191, and 358 ms were used (Fig. 5*C*).

### Offline Analysis

Intrinsic membrane properties (resting membrane potential, membrane capacitance, membrane resistance), voltage clamp step recordings, and evoked PSCs (ePSCs) were analyzed in pClamp10 (Molecular Devices). Action potential features and waveforms were analyzed using Intrinsic Physiology Feature Extractor (IPFX) [©2020 Allen Institute for Brain Science. Intrinsic Physiology Feature Extractor (IPFX). Available from: github.com/AllenInstitute/ipfx] as described elsewhere (28). We made a few modifications in the action potential detection settings. Briefly, action potentials were located when d*V*/d*T* threshold crossings of 20 mV/s. Next, the detected action potentials were approved based on the event height (minimum 20 mV) and width (maximum 10 ms for immature *P8* neurons, and maximum 3 ms for *P18* and *P70* neurons). Action potential half-width was measured as the width (ms) of an action potential at half its amplitude (firing threshold-peak). Interspike interval (ISI) was the time from the baseline of the first action potential to the baseline of the second action potential. Action potential threshold was the baseline voltage of the first action potential and was defined to be 5% of the maximum d*V*/d*t* of the action potential, and the rheobase was the accompanying current that elicited the first action potential. For the firing rates, the number of spikes during the 1 s of step current stimulation were counted. The amplitudes of ePSCs were taken from the PSC generated by the first pulse across all the ISIs presented. The fitted time constants (tau/τ) were taken from the ePSC generated from the first pulse at 50 ms ISI. The time constants were determined by fitting the best number of terms to the decaying part of the ePSC using the following equations:

$$f\left(t\right)=C+\sum_{i=1}^{n}\left(A_{i}e^{-t/\tau_{i}}\right),$$

$$\text{Weighted τ}=\frac{A1\times \text{τ}1}{A1+A2+\ldots An}+\frac{A2\times \text{τ}2}{A1+A2+\ldots An}+\;\frac{An\times \text{τ}n}{A1+A2+\ldots An}.$$

The paired-pulse ratio for ePSCs was calculated as the amplitude of the second ePSC divided by the amplitude of the first ePSC. The frequencies and amplitudes of sPSCs and mPSCs were analyzed in MiniAnalysis (Synaptosoft, Fort Lee, NJ). A minimum amplitude of 5 pA was set as criteria for sPSCs and mPSCs. Data analyses were performed with pClamp10.4 (Molecular Devices), MiniAnalysis software (Synaptosoft, Fort Lee, NJ), and/or Microsoft Excel 2010 (Microsoft Corp.).

### Data Presentation and Statistics

Graphs were generated with GraphPad (Prism 8.3.0 for Windows, GraphPad Software, San Diego, CA). Sample recording traces and images were created in pClamp and Biorender.com. Statistical analyses were conducted using IBM SPSS Statistics for Windows, Version 20.0 (IBM Corp., Armonk, NY). The researcher was blinded to animal genotypes during the experiments and data analysis. The Shapiro–Wilk test of normal distribution was conducted to decide the tests of statistical analyses. The Kruskal–Wallis or Mann–Whitney tests were used to compare non-normally distributed measures. For normally distributed data, statistical tests included independent samples two-tailed *t* tests, one-way analysis of variance (ANOVA), and two-way analysis of variance (ANOVA), and two-way or three-way repeated-measures analysis of variance (RM-ANOVA), as well as post hoc one-way RM ANOVAs and Bonferroni-corrected *t* tests. Equality of variance was assessed using Levene’s test for independent samples *t* tests, and Mauchly’s test of sphericity for RM ANOVAs where Greenhouse Geisser was used to correct degrees of freedom. Outlier analysis was done in GraphPad, and outliers were excluded based on this analysis for the following measures: ePSC amplitudes and weighted τs at *P18* and *P70*. Statistically significant differences were set as *P* values being less than α = 0.05. Exact *P* values are reported, except in cases where *P* < 0.001. See statistics Supplemental Table S1; https://doi.org./10.6084/m9.figshare.21409758 for complete statistical reporting.

## RESULTS

### Age-Related Changes in Intrinsic Properties of Cortical Auditory Neurons

To investigate if the intrinsic properties of neurons are altered due to the loss of *Cntnap2* during development, passive properties such as the resting membrane potential and membrane resistance were measured at *postnatal days* (*P*) *8*–*12*, *P18*–*21*, and *P70*–*90*. Resting membrane potential was not different between the genotypes at all ages (see Supplemental Table S1) but there was a developmental decrease of resting membrane potential (effect of age: [χ^2^(2) = 43.66, *P* < 0.001], with resting potential being significantly more negative at *P18* (*P* < 0.001) and *P70* (*P* < 0.001; Fig. 1*A*), compared with that of *P8*. In parallel, membrane capacitance increased during development [effect of age: *F*(2,145) = 15.269, *P* < 0.001], as it was significantly higher at *P18* (*P*_Bonf_ < 0.001) and *P70* (*P*_Bonf_ < 0.001) compared with *P8* (Fig. 1*B*), but there was no effect of genotype [*F*(1,145) = 0.272, *P* = 0.603]. Membrane resistance also decreased during development [effect of age: χ^2^(2) = 42.814, *P* < 0.001], being significantly lower in *P18* (*P* < 0.001) and *P70* (*P* < 0.001; Fig. 1*C*), compared with *P8*. Membrane resistance was higher in *Cntnap2^−^*^/−^ neurons at *P18* (*U* = 473.5, *P* = 0.016) but lower at *P70* (*U* = 97, *P* = 0.005) as compared with wildtypes. In summary, we observed typical developmental changes of passive properties of neurons, such as a decrease in membrane potential, an increase in cell membrane capacitance, and a decrease in cell membrane resistance, which reflect the maturation of neurons and neuronal networks. The only differences between genotypes were slightly higher cell membrane resistance at *P18* and lower membrane resistance at *P70* in neurons of *Cntnap2^−^*^/−^ animals.

### *Cntnap2^−^*^/−^ Neurons Are More Excitable at P18

Next, we assessed active membrane properties and excitability through action potential features and firing profiles in the current-clamp mode (Fig. 2*A*). Firing thresholds increased during development in all animals regardless of genotype [effect of age: χ^2^(2) = 50.951, *P* < 0.001; no effect of genotype at all ages: see Supplemental Table S1], and it was significantly higher at *P70* than at *P8* (*P* < 0.001; Fig. 2*B*). Likewise, the rheobase current increased during development [effect of age: χ^2^(2) = 93.554, *P* < 0.001], and post hoc pairwise comparisons showed a significant increase at *P18* (*P* < 0.001), as well as *P70* (*P* < 0.001; Fig. 2*C*) compared with *P8*. In addition, rheobase currents were significantly lower in *Cntnap2^−^*^/−^ cells than in wildtypes at *P18* (*U* = 262, *P* = 0.001).

In parallel, there was a significant effect of age on action potential half-width [χ^2^(2) = 63.911 *P* < 0.001; Fig. 3*A*): the half-widths of action potentials significantly decreased between *P8* and *P18* (*P* < 0.001; Fig. 3*B*). Again, there was a genotype effect only at *P18*, where the action potential half-width in *Cntnap2^−^*^/−^ neurons were significantly lower than in wildtype cells (*U =* 172.5, *P* < 0.001). Interspike intervals between the first and second action potentials were not different between the genotypes (see Supplemental Table S1) but were different between age groups [effect of age: χ^2^(2) = 6.760, *P* = 0.034; Fig. 3*C*], where ISIs at *P70* were slightly larger than at *P8* (*P* = 0.033). Neuronal excitability was further assessed through the spiking profiles in response to increasing current injections. *P8* neurons were too immature to maintain spiking activity, as evidenced by their inability to repeatedly fire in response to increasing current injections, although knockout neurons seemed to slightly better respond to the larger depolarizations (Fig. 3, *D* and *G*). At *P18*, knockout neurons exhibited higher firing frequencies for all depolarizations that elicited action potentials (0.12 pA–0.44 pA: *P*_Bonf_ < 0.001; Fig. 3, *E* and *H*), whereas at *P70* there were no significant differences between the wildtype and *Cntnap2^−^*^/−^ firing profiles [*F*(1,39) = 1.419, *P* = 0.241; Fig. 3, *F* and *I*).

### Synaptic Activity Is Altered in Auditory Cortex Neurons with a Functional Loss of *Cntnap2*

**Figure 4.**
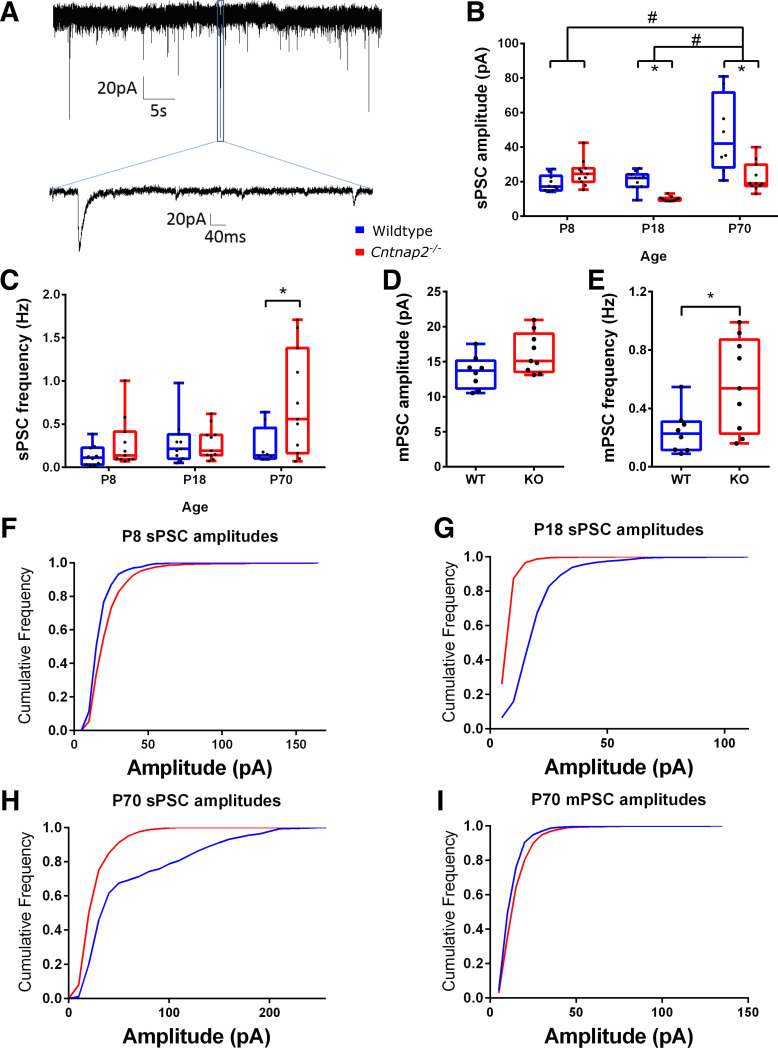
Spontaneous and miniature excitatory postsynaptic potentials show genotypic- and age-related changes in amplitudes and frequency. *A*: sample recording of spontaneous postsynaptic currents (sPSCs) with the amplitudes (*B*) and sPSC frequencies plotted across age groups (*C*). Miniature PSC (mPSC) amplitudes (*D*) and mPSC frequencies (*E*) were recorded while administering tetrodotoxin (TTX) at ages *postnatal* (*P*)*70*–*90*. *F*–*H*: cumulative frequency graphs of amplitudes of sPSCs at *P8* (*F*), *P18* (*G*), and *P70* (*H*), and of mPSCs at *P70* (*I*). Graphs show means ± SE. *Significant differences between genotypes, *P* < 0.05. #Significant differences between age groups, *P* < 0.05.

**Figure 5.**
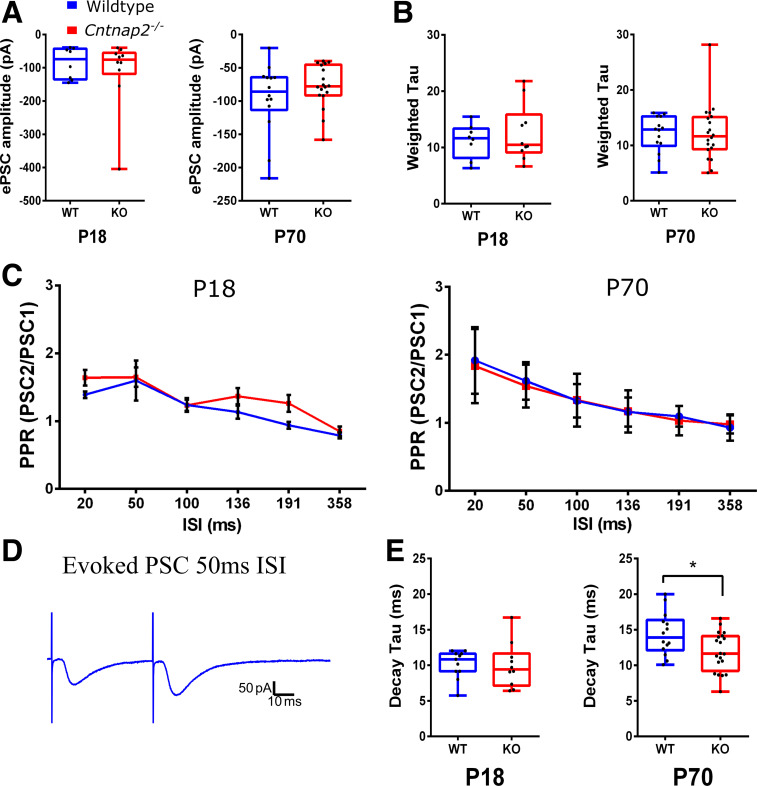
Evoked excitatory postsynaptic potentials (ePSCs) show changes in duration, but not in paired pulse ratio (PPR). Amplitudes of ePSCs after the first stimulus at *postnatal age* (*P*)*18* (*left*) and *P70* (*right*) (*A*), and the weighted τ of the same ePSCs (decay phase) at *P18* (*left*) and *P70* (*right*) (*B*). *C*: paired-pulse ratios (PSC2 amplitude/PSC1 amplitude) at *P18* (*left*) and *P70* (*right*). *D*: sample recordings of ePSCs to paired pulse stimuli with 50 ms interstimulus intervals. *E*: decay τ (time from 100% amplitude to 37% amplitude of the same PSCs in *B* and *C*) at *P18* (*left*) and *P70* (*right*). Graphs show means ± SE. *Significant differences between genotypes, *P* < 0.05.

To investigate if synaptic activity is altered as a result of the loss of *Cntnap2*, we assessed spontaneous, miniature, and evoked excitatory postsynaptic potentials (PSCs, Fig. 4*A* and 5*D*). Spontaneous PSC (sPSCs) were different between the genotypes and the ages as assessed by the sPSC amplitudes [2-way ANOVA, main effect of age: *F*(2,55) = 21.801, *P* < 0.001; main effect of genotype: *F*(1,55) = 14.137, *P* < 0.001; Fig. 4*B*] and frequencies (Independent samples Kruskal–Wallis Test, no effect of age: χ^2^(2) = 5.612, *P* = 0.060; effect of genotype at *P70*, Mann–Whitney test: *U* = 22, *P* = 0.045; Fig. 4*C*). Our analysis revealed that sPSC amplitudes changed differentially in *Cntnap2^−/−^* cells across the three age stages, compared with wildtype cells [2-way ANOVA, significant interaction of age × genotype: *F*(2,55) = 11.577, *P* < 0.001]. Notably at *P70*, sPSC in *Cntnap2^−^*^/−^ cells had smaller amplitudes (*P*_Bonf_ < 0.001, Fig. 4*B*), but higher frequencies (*P*_Bonf_ = 0.002; Fig. 4*C*). Likewise, mPSCs in *Cntnap2^−^*^/−^ cells also occurred at higher frequencies (*P* = 0.02; Fig. 4*E*), whereas amplitudes were not significantly different (*P* = 0.054; Fig. 4*D*). The higher frequencies of both spontaneous and miniature PSCs in the *Cntnap2^−^*^/−^ cells at *P70* may indicate a higher number of synapses providing input to the recorded neuron, and/or a higher pre-synaptic glutamate release probability at the existing synapses. Furthermore, the smaller sPSC amplitudes indicate either weaker synaptic input, due to either decreased neurotransmitter release from presynaptic sides or decreased postsynaptic sensitivity. Since mPSC amplitudes are unchanged, the latter can be ruled out. Therefore, we can conclude from measuring mPSCs and sPSCs that *Cntnap2^−^*^/−^ neurons receive more frequent, but weaker, synaptic input.

Evoked PSCs (ePSCs), which were elicited by stimulating axons in cortical layers 5/6, were recorded in animals at *P18* and *P70* to further shed light on the release probabilities of the synapses (Fig. 5*D*). The amplitudes of the ePSCs were not different in *Cntnap2^−^*^/−^ cells compared with wildtype cells at *P18* (Mann–Whitney test, *U* = 44, *P* = 0.722) or *P70* (Mann–Whitney test, *U* = 127, *P* = 0.649; Fig. 5*A*). Moreover, the paired-pulse ratio (PPR) was assessed to gain further insight into potential changes of synaptic release probability. Both genotypes showed strong paired-pulse facilitation at short ISIs with a main effect of ISI (3-way repeated measures ANOVA, 3.277, 150.751) = 66.307, *P* < 0.001; Fig. 5*C*), no main effect of age [3-way repeated measures ANOVA, *F*(1,46) = 1.602, *P* = 0.212], and no main effect of genotype [3-way repeated measures ANOVA, *F*(1,46) = 0.235, *P* = 0.630]. This indicates that there is no difference in synaptic release probability between genotypes, which leaves a higher number of synapses providing synaptic input to *Cntnap2^−^*^/−^ cells as a possible explanation for the increased frequency of sPSC. The time constants of the decaying phase of ePSCs (fitted to 100% − 37% of maximum PSC amplitude, weighted τ) were not different between genotypes [Independent-samples 2-tailed *t* test, *P18*: *t*(16) = 0.092, *P* = 0.928; and Mann–Whitney test, *P70*: *U* = 148, *P* = 0.780; Fig. 5*B*]. However, τ was smaller in *Cntnap2^−^*^/−^ cells at *P70* (Mann–Whitney test, *U* = 197, *P* = 0.047; Fig. 5*E*), but it was not different between the genotypes at *P18* [*t*(16) = 0.682, *P* = 0.505].

## DISCUSSION

In this study, we examined intrinsic membrane properties as well as postsynaptic currents in cortical neurons of the primary auditory cortex of *Cntnap2^−^*^/−^ rats and their wildtype littermates through crucial stages of postnatal development, to see to what extent reported alterations in auditory processing in the adult might potentially be caused by changes in the developmental trajectory. We focused on an early stage (*P8*–*12*) where the meatus of the ears are still closed and hearing is limited (29), a juvenile stage (*P18*–*21*) where hearing input leads to massive synaptic rewiring and synaptic pruning (30, 31), and an adult stage (*P70*–*90*).

### Developmental Changes

As expected, we observed a number of developmental changes between age stages that mainly reflect the maturation of immature cortical neurons. This includes a decrease in resting membrane potential and membrane resistance, an increase in cell capacitance, as well as an increase in firing threshold and rheobase, while the action-potential half-width decreased. All of these changes occurred mostly between *P8* and *P18*, and were accompanied by the establishment of mature firing patterns, i.e., the ability of sustained firing upon a lasting depolarization. In contrast, amplitudes of spontaneous postsynaptic currents (sPSCs) were significantly increased between *P18* and *P70*, indicating changes in synaptic wiring or postsynaptic receptor density after hearing onset in this later stage of development. Moreover, the increase in frequencies of sPSCs could be explained by either a higher release probability and higher spontaneous activity in presynaptic neurons, or a higher connectivity between neurons resulting in more input synapses. An increase in release probability would be expected to reduce paired-pulse facilitation (32, 33). The paired-pulse ratio of evoked EPSC was not different between *P70* compared with *P18*, indicating no change in release probability, which does not explain the increased frequencies of sPSCs at *P70*. Therefore, it is possible that there is a higher connectivity between cortical neurons in *P70* than in *P18*, reflecting the establishment of mature cortical neuronal networks after the onset of hearing. It is important to note that evoked and miniature PSCs may involve different pools of presynaptic vesicles and even different populations of postsynaptic receptors (34, 35), therefore they may have separate mechanisms that are not entirely comparable. It is also worth mentioning that since stimulating in *layers 5*/*6* likely activates a variety of synapse types that have different release dynamics, we should be cautious with the interpretation of the evoked PSC data.

### Differences in *Cntnap2^−^^/−^* Neurons

Genotypic differences in intrinsic membrane properties between wildtype and *Cntnap2^−^*^/−^ animals were mainly observed at *P18*, where a reduced rheobase, reduced half-width of action potentials and a significantly higher firing rate in response to sustained depolarization was observed in *Cntnap2^−^*^/−^ cells. Generally, all of these observations indicate that auditory cortical neurons of *Cntnap2^−^*^/−^ animals are more excitable during this phase of high synaptic plasticity at *P18*. Interestingly, these changes do not extend into the adult; cell excitability seemed to largely normalize by the age of *P70*. In contrast, lasting changes in postsynaptic currents, i.e., higher frequencies in mPSCs and sPSCs, as well as lower amplitudes of sPSCs and shorter ePSC in *Cntnap2^−^*^/−^ cells manifest in the adult stage. Interestingly, the genotypic differences in the amplitudes of sPSCs and mPSCs were not consistent at *P70*. The spontaneous and miniature PSCs differ because mPSCs were measured in the presence of tetrodotoxin, which results in only spontaneously released presynaptic vesicles, whereas sPSC were measured without the presence of tetrodotoxin and therefore we measure a mix of spontaneously released and action potential-driven vesicle releases. Given that sPSC amplitudes were smaller in *Cntnap2^−^*^/−^ cells but there were no differences in mPSC amplitudes, this may indicate that the pools of vesicles involved in action potentials package less neurotransmitter or have less postsynaptic receptor density, whereas these are not changed in vesicles and postsynaptic receptors involved in spontaneous vesicle release. Moreover, given that the frequencies of mPSCs and sPSCs are similarly increased in *Cntnap2^−^*^/−^ cells, this could mean that this hyperactivity is occurring in pools of vesicles involved in spontaneous release rather than those involved in action-potentials. Following the aforementioned interpretation of the developmental changes, this indicates a hyper-connectivity within the auditory cortex of *Cntnap2^−^*^/−^ animals.

Changes in synaptic activity due to *Cntnap2* deletion have been reported before, for example inhibitory synaptic activity was reduced in pyramidal neurons in visual cortices of 6- to 8-wk old *Cntnap2^−^*^/−^ mice, but there were few differences at 3–4 wk of age (19). Similarly, we found that synaptic changes as a result of *Cntnap2* deletion were more robust in the older animals compared with younger. This could be attributed to the fact that synaptic networks are established over age, and so any effects of *Cntnap2* on synaptic remodeling would show up only later in development. Although we demonstrated increased sPSC and mPSC frequencies and decreased sPSCs amplitudes in the *Cntnap2^−^*^/−^ cells, another study has shown both reduced mPSC amplitudes and frequencies in the mPFC of *Cntnap2^−^*^/−^ mice (20). This may mean that *Cntnap2* deletion may not affect all cortical areas equally, as similar studies also found no genotypic differences in the visual cortex at *weeks 3*–*4* (19). These differences could also be due to specific effects of *Cntnap2* deletion on upstream structures in the auditory pathway, which could then be selectively affecting the neuronal activity in the auditory cortex.

Intrinsic differences such as increased action potential half-widths and more depolarized resting membrane potentials have been described in fast-spiking *Cntnap2^−^*^/−^ interneurons in the somatosensory cortex of mice (22). Another study found a reduction of GABAergic neurons in *Cntnap2* null zebrafish (36). Although we did not study inhibitory interneurons in this study, it would be worth investigating to what extent the increased pyramidal neuron excitability at *P18* is potentially a result of changes in interneurons.

Social behavioral deficits have been rescued in *Cntnap2^−^*^/−^ mice by both inhibiting excitability of pyramidal neurons and increasing the excitability of inhibitory interneurons in the medial prefrontal cortices (mPFC) (21). This is in line with our results as we found an increased excitability of pyramidal neurons of *Cntnap2^−^*^/−^ rats, and given that this rat model displays hyper-reactivity to auditory stimuli, perhaps a decrease in the excitation or increase of inhibition in the auditory cortex would rescue this behavior to wildtype levels.

We found that intrinsic membrane properties that were present at *P18* normalize upon adulthood, unlike synaptic connectivity. It is possible that the intrinsic differences at *P18*, which is around the critical age when networks are being established, could be causing long lasting hyper-connectivity in the neural networks. Hyper-connectivity in certain regions of autistic brains has been described before in imaging studies (37–42). Similarly, several rodent models of autism have demonstrated synaptic hyper-connectivity (43–46). The fact that there are more, but weaker synapses could be due to synaptic scaling, an attempt of the network to maintain balance between excitation and inhibition (47–51). However, other studies have also shown hypo-connectivity in certain areas such as in mPFC in the *Cntnap2* mouse model (20), and in certain brain regions in humans (37, 41), again indicating that not all brain areas are affected equally and in the same way. In conclusion, it is unknown to what extent the hyper-connectivity in the adult is caused by the hyperexcitability of neurons at the *P18* stage, but it is possible that these changes are linked.

Many of the results of the adult stage from this study are different to our previous report, where *layers 2*/*3* of auditory cortices in adult homozygously bred *Cntnap2^−^*^/−^ rats were studied (9). In contrast to the present study, adult (*P70*–*90*) *Cntnap2^−^*^/−^ rats from this previous report exhibited persistent alterations in intrinsic membrane properties, such as decreased membrane capacitance, more negative firing threshold, smaller rheobase current, and larger and earlier inward transient currents in response to stimulation, indicating the neurons were more excitable. Action potential properties were also different in *Cntnap2^−^*^/−^ cells, as larger action-potential half-widths and altered firing frequencies were found; all of which were not different in adulthood in the present study. Furthermore, although we saw alterations in sPSCs and mPSCs in the current study, there were no synaptic differences found in the previous study, although paired-pulsed ratios of ePSCs were unchanged in both studies. Finally, whereas there were persistent intrinsic neuron properties differences but no network-level synaptic differences (sPSCs) in the adult homozygously bred rats, we saw the opposite in the heterozygously derived rats in that the intrinsic differences normalize by adult stage yet the synaptic differences persist.

These marked differences between adult cortical neuron properties in the two studies are somewhat surprising, especially because the previous study was conducted under the same experimental conditions and the same experimenter as the current study, with the only difference that the rats in the previous study were derived from homozygous pairings (WT × WT or KO × KO), whereas the animals for the present study were littermates derived from heterozygous pairings. Considering the potential for the breeding strategy, i.e., the fetal and postnatal environment, to significantly influence the cellular properties of cortical neurons, it is worth noting that the maturation of sound processing in the auditory brainstem, as measured as auditory brainstem response (ABR), was also found to be differently affected: homozygously bred *Cntnap2^−^*^/−^ rats showed a delayed development in auditory brainstem responses compared with wildtype controls (15), whereas *Cntnap2^−^*^/−^ rats derived from heterozygous pairings did not (10). It must be noted that while the heterozygously bred animals were littermates, the homozygously bred animals were not from the same family, and therefore genetic drift could contribute to the differences between the genotypes. Furthermore, in another study, we have also observed dramatic differences in ultrasonic vocalizations between heterozygously- versus homozygously derived *Cntnap2^−^*^/−^ offspring from litters that had similar genetic background (52). To further understand how maternal phenotype affects the offspring, an ongoing study is examining how cross-fostering homozygously derived *Cntnap2^−^*^/−^ offspring to heterozygous dams and litters alters the phenotype. Collectively, these findings bring into question to what extent the disruption of the *Cntnap2* gene is sufficient to cause brain alterations, or whether the environment, such as the maternal in utero environment, maternal behavior/offspring care, or interaction with WT versus knockout littermates, can ultimately alter the trajectory of how the gene deletion affects development.

### Conclusion

We describe age- and genotype-related changes in neuron membrane properties and synaptic input in cortical auditory neurons of wildtype and *Cntnap2^−^*^/−^ rats derived from heterozygous breeding. The understanding of these changes throughout the developmental timeline, and how they are potentially caused, are crucial to understand the neuronal basis of altered perception and auditory-evoked behavior observed in ASD. We observed transient changes intrinsic neuronal properties, which were replaced by persisting network changes in the adult. It remains to be determined to what extent these are linked. Furthermore, we show a large influence of breeding scheme on the cellular phenotype, which indicates that environmental aspects may play an important role in the manifestations of *Cntnap2^−^*^/−^-related electrophysiological changes in the developing auditory system. A deeper understanding of the temporal aspects and the influence of genetic versus environmental influences will help us develop time-sensitive interventions that could help alleviate some of the symptoms present in *Cntnap2^−^*^/−^-related developmental syndromes and more generally in ASD.

## DATA AVAILABILITY

Data will be made available upon reasonable request.

## SUPPLEMENTAL DATA

10.6084/m9.figshare.21409758 Supplemental Table S1: https://doi.org./10.6084/m9.figshare.21409758.

## GRANTS

This work was funded by an National Sciences and Engineering Research Council (NSERC) discovery Grant (to S. Schmid) and by a Canadian Institutes of Health Research (CIHR) project Grant (to S. Schmid and B. L. Allman).

## DISCLOSURES

No conflicts of interest, financial or otherwise, are declared by the authors.

## AUTHOR CONTRIBUTIONS

S.S. conceived and designed research; R.S.M. performed experiments; R.S.M. analyzed data; R.S.M., B.L.A., and S.S. interpreted results of experiments; R.S.M. prepared figures; R.S.M. drafted manuscript; B.L.A. and S.S. edited and revised manuscript; B.L.A. and S.S. approved final version of manuscript.

## References

[1] Alarcón M, Abrahams BS, Stone JL, Duvall JA, Perederiy JV, Bomar JM, Sebat J, Wigler M, Martin CL, Ledbetter DH, Nelson SF, Cantor RM, Geschwind DH. Linkage, association, and gene-expression analyses identify CNTNAP2 as an autism-susceptibility gene. Am J Hum Genet 82: 150–159, 2008. doi:10.1016/j.ajhg.2007.09.005. 18179893 PMC2253955

[2] Bakkaloglu B, O'Roak BJ, Louvi A, Gupta AR, Abelson JF, Morgan TM, Chawarska K, Klin A, Ercan-Sencicek AG, Stillman AA, Tanriover G, Abrahams BS, Duvall JA, Robbins EM, Geschwind DH, Biederer T, Gunel M, Lifton RP, State MW. Molecular cytogenetic analysis and resequencing of contactin associated protein-like 2 in autism spectrum disorders. Am J Hum Genet 82: 165–173, 2008. doi:10.1016/j.ajhg.2007.09.017. 18179895 PMC2253974

[3] Arking DE, Cutler DJ, Brune CW, Teslovich TM, West K, Ikeda M, Rea A, Guy M, Lin S, Cook EH, Chakravarti A. A common genetic variant in the neurexin superfamily member CNTNAP2 increases familial risk of autism. Am J Hum Genet 82: 160–164, 2008. doi:10.1016/j.ajhg.2007.09.015. 18179894 PMC2253968

[4] Rodenas-Cuadrado P, Pietrafusa N, Francavilla T, La Neve A, Striano P, Vernes SC. Characterisation of CASPR2 deficiency disorder—a syndrome involving autism, epilepsy and language impairment. BMC Med Genet 17: 8, 2016. doi:10.1186/s12881-015-0265-z. 26843181 PMC4739328

[5] Strauss KA, Puffenberger EG, Huentelman MJ, Gottlieb S, Dobrin SE, Parod JM, Stephan DA, Morton DH. Recessive symptomatic focal epilepsy and mutant contactin-associated protein-like 2. N Engl J Med 354: 1370–1377, 2006. doi:10.1056/NEJMoa052773. 16571880

[6] Whitehouse AJO, Bishop DVM, Ang QW, Pennell CE, Fisher SE. CNTNAP2 variants affect early language development in the general population. Genes Brain Behav 10: 451–456, 2011. [Erratum in *Genes Brain Behav* 11: 501, 2012]. doi:10.1111/j.1601-183X.2011.00684.x. 21310003 PMC3130139

[7] Gdalyahu A, Lazaro M, Penagarikano O, Golshani P, Trachtenberg JT, Gescwind DH. The autism related protein contactin-associated protein-like 2 (CNTNAP2) stabilizes new spines: An in vivo mouse study. PLoS One 10: e0125633, 2015. doi:10.1371/journal.pone.0125633. 25951243 PMC4423902

[8] Poliak S, Peles E. The local differentiation of myelinated axons at nodes of Ranvier. Nat Rev Neurosci 4: 968–980, 2003. doi:10.1038/nrn1253. 14682359

[9] Scott KE, Mann RS, Schormans AL, Schmid S, Allman BL. Hyperexcitable and immature-like neuronal activity in the auditory cortex of adult rats lacking the language-linked CNTNAP2 gene. Cereb Cortex 32: 4797–4817, 2022. doi:10.1093/cercor/bhab517. 35106542 PMC9626820

[10] Zheng A, Scott KE, Schormans AL, Mann R, Allman BL, Schmid S. Differences in startle and prepulse inhibition in contactin-associated protein-like 2 knock-out rats are associated with sex-specific alterations in brainstem neural activity. Neuroscience 513: 96–110, 2023. doi:10.1016/j.neuroscience.2023.01.020. 36708798

[11] Condro MC, White SA. Distribution of language-related Cntnap2 protein in neural circuits critical for vocal learning. J Comp Neurol 522: 169–185, 2014. doi:10.1002/cne.23394. 23818387 PMC3883908

[12] Gordon A, Adamsky K, Vainshtein A, Frechter S, Dupree JL, Rosenbluth J, Peles E. Caspr and Caspr2 are required for both radial and longitudinal organization of myelinated axons. J Neurosci 34: 14820–14826, 2014. doi:10.1523/JNEUROSCI.3369-14.2014. 25378149 PMC4220019

[13] Peñagarikano O, Abrahams BS, Herman EI, Winden KD, Gdalyahu A, Dong H, Sonnenblick LI, Gruver R, Almajano J, Bragin A, Golshani P, Trachtenberg JT, Peles E, Geschwind DH. Absence of CNTNAP2 leads to epilepsy, neuronal migration abnormalities, and core autism-related deficits. Cell 147: 235–246, 2011. doi:10.1016/j.cell.2011.08.040. 21962519 PMC3390029

[14] Scott KE, Kazazian K, Mann RS, Möhrle D, Schormans AL, Schmid S, Allman BL. Loss of Cntnap2 in the rat causes autism-related alterations in social interactions, stereotypic behavior, and sensory processing. Autism Res 13: 1698–1717, 2020. doi:10.1002/aur.2364. 32918359

[15] Scott KE, Schormans AL, Pacoli KY, Oliveira CD, Allman BL, Schmid S. Altered auditory processing, filtering, and reactivity in the cntnap2 knock-out rat model for neurodevelopmental disorders. J Neurosci 38: 8588–8604, 2018. doi:10.1523/JNEUROSCI.0759-18.2018. 30126973 PMC6596223

[16] Truong DT, Rendall AR, Castelluccio BC, Eigsti IM, Holly R. Auditory processing and morphological anomalies in medial geniculate nucleus of Cntnap2 mutant mice. Behav Neurosci 129: 731–741, 2015. doi:10.1037/bne0000096. 26501174

[17] Anderson GR, Galfin T, Xu W, Aoto J, Malenka RC, Sudhof TC. Candidate autism gene screen identifies critical role for cell-adhesion molecule CASPR2 in dendritic arborization and spine development. Proc Natl Acad Sci USA 109: 18120–18125, 2012. doi:10.1073/pnas.1216398109. 23074245 PMC3497786

[18] Varea O, Martin-de-Saavedra MD, Kopeikina KJ, Schürmann B, Fleming HJ, Fawcett-Patel JM, Bach A, Jang S, Peles E, Kim E, Penzes P. Synaptic abnormalities and cytoplasmic glutamate receptor aggregates in contactin associated protein-like 2/Caspr2 knockout neurons. Proc Natl Acad Sci USA 112: 6176–6181, 2015. doi:10.1073/pnas.1423205112. 25918374 PMC4434727

[19] Bridi MS, Park SM, Huang S. Developmental disruption of GABA _A_ R-meditated inhibition in Cntnap2 KO mice. eNeuro 4: ENEURO.0162-17.2017, 2017. doi:10.1523/ENEURO.0162-17.2017. 28966979 PMC5617210

[20] Lazaro MT, Taxidis J, Shuman T, Bachmutsky I, Ikrar T, Santos R, Marcello GM, Mylavarapu A, Chandra S, Foreman A, Goli R, Tran D, Sharma N, Azhdam M, Dong H, Choe KY, Peñagarikano O, Masmanidis SC, Rácz B, Xu X, Geschwind DH, Golshani P. Reduced prefrontal synaptic connectivity and disturbed oscillatory population dynamics in the CNTNAP2 model of autism. Cell Rep 27: 2567–2578.e6, 2019. doi:10.1016/j.celrep.2019.05.006. 31141683 PMC6553483

[21] Selimbeyoglu A, Kim CK, Inoue M, Lee SY, Hong ASO, Kauvar I, Ramakrishnan C, Fenno LE, Davidson TJ, Wright M, Deisseroth K. Modulation of prefrontal cortex excitation/inhibition balance rescues social behavior in CNTNAP2-deficient mice. Sci Transl Med 9: eaah6733, 2017. doi:10.1126/scitranslmed.aah6733. 28768803 PMC5723386

[22] Vogt D, Cho KKA, Shelton SM, Paul A, Huang ZJ, Sohal VS, Rubenstein JLR. Mouse Cntnap2 and human CNTNAP2 ASD alleles cell autonomously regulate PV+ cortical interneurons. Cereb Cortex 28: 13868–3879, 2017. doi:10.1093/cercor/bhx248. 29028946 PMC6455910

[23] Gordon A, Salomon D, Barak N, Pen Y, Tsoory M, Kimchi T, Peles E. Expression of Cntnap2 (Caspr2) in multiple levels of sensory systems. Mol Cell Neurosci 70: 42–53, 2016. doi:10.1016/j.mcn.2015.11.012. 26647347

[24] Bosch D, Schmid S. Activation of muscarinic cholinergic receptors inhibits giant neurones in the caudal pontine reticular nucleus. Eur J Neurosci 24: 1967–1975, 2006. doi:10.1111/j.1460-9568.2006.05085.x. 17040474

[25] Simons-Weidenmaier NS, Weber M, Plappert CF, Pilz PKD, Schmid S. Synaptic depression and short-term habituation are located in the sensory part of the mammalian startle pathway. BMC Neurosci 7: 1–13, 2006. doi:10.1186/1471-2202-7-1. 16684348 PMC1479352

[26] Zaman T, De Oliveira C, Smoka M, Narla C, Poulter MO, Schmid S. BK channels mediate synaptic plasticity underlying habituation in rats. J Neurosci 37: 4540–4551, 2017. doi:10.1523/JNEUROSCI.3699-16.2017. 28348135 PMC6596664

[27] Marino M, Misuri L, Brogioli D. A new open source software for the calculation of the liquid junction potential between two solutions according to the stationary Nernst-Planck equation. arXiv, 2014. doi:10.48550/ARXIV.1403.3640.

[28] Gouwens NW, Sorensen SA, Berg J, Lee C, Jarsky T, Ting J, et al Classification of electrophysiological and morphological neuron types in the mouse visual cortex. Nat Neurosci 22: 1182–1195, 2019. doi:10.1038/s41593-019-0417-0. 31209381 PMC8078853

[29] Geal-Dor M, Freeman S, Li G, Sohmer H. Development of hearing in neonatal rats: air and bone conducted ABR thresholds. Hear Res 69: 236–242, 1993. doi:10.1016/0378-5955(93)90113-f. 8226345

[30] Juraska JM, Drzewiecki CM. Cortical reorganization during adolescence: what the rat can tell us about the cellular basis. Dev Cogn Neurosci 45: 100857, 2020. doi:10.1016/j.dcn.2020.100857. 32927244 PMC7495017

[31] Takesian AE, Kotak VC, Sanes DH. Developmental hearing loss disrupts synaptic inhibition: Implications for auditory processing. Future Neurol 4: 331–349, 2009. doi:10.2217/FNL.09.5. 20161214 PMC2716048

[32] Higley MJ, Rosenberg BJ, Levy AD, Xiao X, Koleske AJ. Disruption of coordinated presynaptic and postsynaptic maturation underlies the defects in hippocampal synapse stability and plasticity in Abl2/Arg-deficient mice. J Neurosci 36: 6778–6791, 2016. doi:10.1523/JNEUROSCI.4092-15.2016. 27335408 PMC4916252

[33] Zucker RS, Regehr WG. Short term synaptic plasticity. Annu Rev Physiol 64: 355–405, 2002. doi:10.1146/annurev.physiol.64.092501.114547. 11826273

[34] Atasoy D, Ertunc M, Moulder KL, Blackwell J, Chung C, Su J, Kavalali ET. Spontaneous and evoked glutamate release activates two populations of NMDA receptors with limited overlap. J Neurosci 28: 10151–10166, 2008. doi:10.1523/JNEUROSCI.2432-08.2008. 18829973 PMC2578837

[35] Kavalali ET. The mechanisms and functions of spontaneous neurotransmitter release. Nat Rev Neurosci 16: 5–16, 2015. doi:10.1038/nrn3875. 25524119

[36] Hoffman EJ, Turner KJ, Fernandez JM, Rihel J, State MW, Giraldez AJ, Hoffman EJ, Turner KJ, Fernandez JM, Cifuentes D, Ghosh M, Ijaz S. Estrogens suppress a behavioral phenotype in zebrafish mutants of the autism risk gene, CNTNAP2. Neuron 89: 725–733, 2016. doi:10.1016/j.neuron.2015.12.039. 26833134 PMC4766582

[37] Iidaka T, Kogata T, Mano Y, Komeda H. Thalamocortical hyperconnectivity and amygdala-cortical hypoconnectivity in male patients with autism spectrum disorder. Front Psychiatry 10: 1–11, 2019. doi:10.3389/fpsyt.2019.00001. 31057443 PMC6482335

[38] Orekhova EV, Elsabbagh M, Jones EJ, Dawson G, Charman T, Baron-Cohen S, Bedford R, Bolton P, Fernandes J, Ganea N, Garwood H, Gliga T, Hudry K, Murias M, Ribeiro H, Tucker L, Volein A, Webb SJ. EEG hyper-connectivity in high-risk infants is associated with later autism. J Neurodev Disord 6: 1–11, 2014. doi:10.1186/1866-1955-6-1. 25400705 PMC4232695

[39] Seghatol-Eslami VC, Maximo JO, Ammons CJ, Libero LE, Kana RK. Hyperconnectivity of social brain networks in autism during action-intention judgment. Neuropsychologia 137: 107303, 2020. doi:10.1016/j.neuropsychologia.2019.107303. 31837376

[40] Supekar K, Uddin LQ, Khouzam A, Phillips J, Gaillard WD, Kenworthy LE, Yerys BE, Vaidya CJ, Menon V. Brain hyper-connectivity in children with autism and its links to social deficits. Cell Rep 5: 738–747, 2013. doi:10.1016/j.celrep.2013.10.001. 24210821 PMC3894787

[41] Xu J, Wang H, Zhang L, Xu Z, Li T, Zhou Z, Zhou Z, Gan Y, Hu Q. Both hypo-connectivity and hyper-connectivity of the insular subregions associated with severity in children with autism spectrum disorders. Front Neurosci 12: 1–9, 2018. doi:10.3389/fnins.2018.00001. 29695950 PMC5904282

[42] Zeeland AAS, Abrahams BS, Alvarez-Retuerto AI, Sonnenblick LI, Rudie JD, Ghahremani D, Mumford JA, Poldrack RA, Dapretto M, Geschwind DH, Bookheimer SY. Altered functional connectivity in frontal lobe circuits is associated with variation in the autism risk gene CNTNAP2. Sci Transl Med 2: 56ra80, 2011. doi:10.1126/scitranslmed.3001344. 21048216 PMC3065863

[43] Nagode DA, Meng X, Winkowski E, Kareddy V, Kao JPY, Kanold PO, Nagode DA, Meng X, Winkowski DE, Smith E, Khan-Tareen H, Kareddy V. Abnormal development of the earliest cortical circuits in a mouse model of autism spectrum disorder. Cell Rep 18: 1100–1108, 2017. doi:10.1016/j.celrep.2017.01.006. 28147267 PMC5488290

[44] Rinaldi T, Silberberg G, Markram H. Hyperconnectivity of local neocortical microcircuitry induced by prenatal exposure to valproic acid. Cereb Cortex 18: 763–770, 2008. doi:10.1093/cercor/bhm117. 17638926

[45] Testa-Silva G, Loebel A, Giugliano M, Kock CD, Mansvelder HD, Meredith M. Hyperconnectivity and slow synapses during early development of medial prefrontal cortex in a mouse model for mental retardation and autism. Cerebral Cortex 22: 1333–1342, 2012. doi:10.1093/cercor/bhr224. 21856714 PMC3561643

[46] Zaslavsky K, Zhang W, Mccready FP, Rodrigues DC, Deneault E, Loo C, Zhao M, Ross PJ, Hajjar JE, Romm A, Thompson T, Piekna A, Wei W, Wang Z, Khattak S, Mufteev M, Pasceri P, Scherer SW, Salter MW, Ellis J. SHANK2 mutations associated with autism spectrum disorder cause hyperconnectivity of human neurons. Nat Neurosci 22: 556–564, 2019. doi:10.1038/s41593-019-0365-8. 30911184 PMC6475597

[47] Bassi MS, Iezzi E, Gilio L, Centonze D, Buttari F. Synaptic plasticity shapes brain connectivity: implications for network topology. Int J Mol Sci 20: 6193, 2019. doi:10.3390/ijms20246193. 31817968 PMC6940892

[48] Desai NS, Cudmore RH, Nelson SB, Turrigiano GG. Critical periods for experience- dependent synaptic scaling in visual cortex. Nat Neurosci 5: 783–789, 2002. doi:10.1038/nn878. 12080341

[49] Shepherd JD, Rumbaugh G, Wu J, Chowdhury S, Plath N, Kuhl D, Huganir RL, Worley PF. Arc/Arg3.1 mediates homeostatic synaptic scaling of AMPA receptors. Neuron 52: 475–484, 2006. doi:10.1016/j.neuron.2006.08.034. 17088213 PMC1764219

[50] Turrigiano G. Homeostatic synaptic plasticity: local and global mechanisms for stabilizing neuronal function. Cold Spring Harb Perspect Biol 4: a005736, 2012. doi:10.1101/cshperspect.a005736. 22086977 PMC3249629

[51] Turrigiano GG, Leslie KR, Desai NS, Rutherford LC, Nelson SB. Activity-dependent scaling of quantal amplitude in neocortical neurons. Nature 391: 892–896, 1998. doi:10.1038/36103. 9495341

[52] Möhrle D, Yuen M, Zheng A, Haddad FL, Allman BL, Schmid S. Characterizing maternal isolation-induced ultrasonic vocalizations in a gene–environment interaction rat model for autism. Genes Brain Behav e12841, 2023. doi:10.1111/gbb.12841. 36751016 PMC10242206

